# Genetic characterization and risk factors for feline hemoplasma infection in semi-domesticated cats in Bangkok, Thailand

**DOI:** 10.14202/vetworld.2020.975-980

**Published:** 2020-05-23

**Authors:** Thom Do, Ketsarin Kamyingkird, Linh Khanh Bui, Tawin Inpankaew

**Affiliations:** 1 Department of Parasitology, Faculty of Veterinary Medicine, Kasetsart University, Bangkok, Thailand; 2 Department of Parasitology, Faculty of Veterinary Medicine, Vietnam National University of Agriculture, Hanoi, Vietnam

**Keywords:** hemoplasma, semi-domesticated cat, Thailand, vector-borne diseases

## Abstract

**Background and Aim:**

Stray cats are a reservoir for various zoonotic diseases relevant to public health in Thailand. The vector-borne infection, hemoplasmosis, also known as infectious anemia, is one such disease carried by domestic and wild felids. This study focuses on molecular characterization and phylogenetic analysis of hemoplasma in semi-domesticated cats in Bangkok, Thailand.

**Materials and Methods:**

In total, 473 blood samples were collected from 53 temple communities in 34 metropolitan districts and assayed using polymerase chain reaction (PCR) to amplify partial 16S rRNA genes for hemoplasma detection. Risk factors for hemoplasma infection (gender, age, free-roaming, and ectoparasite exposure) were analyzed using Chi-square tests, logistic regression, and odds ratios (OR) with 95% confidence intervals (95% CI) using R software (version 3.6.1). A phylogenetic tree was established from genetic characterization of hemoplasmas.

**Results:**

In total, 180 samples (38.05%) were positive for hemoplasma. Of half of the positive sequenced samples, 83.33% were infected with *Candidatus* Mycoplasma haemominutum (*CMhm*), 13.33% with *Mycoplasma haemofelis* (*Mhf*), and 3.33% with *Candidatus* Mycoplasma turicensis (*CMt*). Cats over 5 years old were more likely to be infected than younger cats (p<0.005, OR=3.8, 95% CI=1.64-4.78). Cats were diagnosed as positive based on PCR assays in 97% (33/34) of districts surveyed. The phylogenetic tree showed two majority clusters with three clades of feline hemotropic mycoplasma.

**Conclusions:**

Overall, the survey shows the prevalence (38.05%) and distribution of feline hemoplasma in semi-domesticated cats. This information will contribute to effective prevention and control strategies to minimize infections by feline vector-borne pathogens in Thailand.

## Introduction

Hemotropic mycoplasmas (also known as hemoplasmas) are parasitic bacteria without cell walls. These organisms are related to mollicutes and are unculturable. The pathogens may be present in the blood of mammals, such as cats and dogs. Infections are caused by hemoplasmas attachment and growth on surfaces of red blood cells [1]. At least three common species of feline hemoplasmas are found in domestic cats: *Mycoplasma haemofelis* (*Mhf*), *Candidatus* Mycoplasma haemominutum (*CMhm*), and *Candidatus* Mycoplasma turicensis (*CMt*) [2]. A recent study from Chile suggests that cats may also be infected with the canine hemoplasmas-like organism, *Candidatus* Mycoplasma hematoparvum [3]. These species may be important primary causes of hemolytic anemia in felids with distinct pathogenicity [1,4]; however, other studies suggest that hemoplasmas are more opportunistic pathogens [5].

*Mhf*-infected cats often display hemolysis and severe anemia, and *CMhm-*infected and *CMt*-infected cats show other non-specific clinical signs, such as pallor, anorexia, weakness, weight loss, depression, and dehydration [6]. Feline hemoplasmas may infect humans, based on a molecular study of an *Mhf-*like organism detected in an HIV-positive, immunocompromised patient [7]. Bloodsucking arthropods likely play a role feline hemoplasmas transmission. Studies report feline hemoplasmas infection in fleas collected from cats [8-10] and some ticks [11]. The vector for transmission may be the cat flea, *Ctenocephalides felis*, as demonstrated in experimental *Mhf* infection [1,11]. Direct transmission, by aggressive feline or interspecies interactions, may also play a role in transmission [6].

Since hemoplasma cannot be cultured, “classical” diagnosis of feline hemoplasma was previously based on microscopic observation of blood smears using Romanowsky-type stains; however, this method has poor sensitivity (0-37.5%) and specificity (84-98%) [6,12]. Polymerase chain reaction (PCR) is more sensitive than cytology for hemoplasmas detection [12-14], and PCR-based tests described to date are based on amplification of segments of the hemoplasma16S RNA gene [6]. Molecular techniques have been applied widely in studies of feline hemoplasmosis prevalence with incidence ranging from 9% to 16% in Italy, Denmark, Spain, and Germany (European) [1,15-17] to 26-47% in Japan, Korea, and Iran (Asian) [18-20]. In Thailand, numbers of abandoned, unwanted stray animals at monasteries and other public areas such as parks, schools, or alongside roads are substantial and continue to grow where no control programs are in place [21]. These animals have a close relationship to people and close contact with indoor pets that occasionally run free outdoors. Pets may put their owners at risk by bringing hemoplasmosis into their home. Transmission to human is theoretically possible [22].

This study was conducted utilizing partial 16S rRNA gene-based PCR assays to investigate feline hemoplasma species in stray cats in Bangkok, Thailand, and, simultaneously, identify risk factors associated with infection in the study area. The current findings are important for future surveillance and for informing diagnosis and control of feline disease in Thailand.

## Materials and Methods

### Ethical approval

Approval for this study was obtained from the Animal Ethics Committee of Kasetsart University, Bangkok, Thailand (ACKU60-VET006). Informed consent forms were signed by monks, nuns, and animal caretakers before samples were collected.

### Study areas and study period

**Figure-1:**
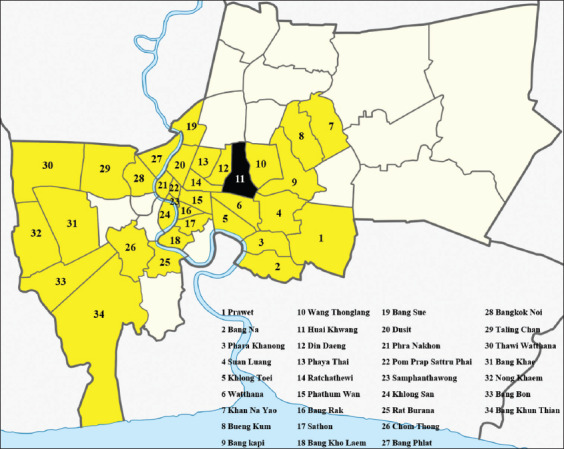
Map of study area in Bangkok. The highlighting 34 selected districts (yellow-colored areas identified as infected with the pathogen and black-colored area indicates no detected of infection) (https://upload.wikimedia.org/wikipedia/commons/4/4f/Thailand_Bangkok_location_map.png).

This randomized cross-sectional study was conducted in 53 Buddhist temples located in 34 districts of the Bangkok metropolitan area during spring and summer months between March and June 2017 (Figure-1). In this investigation, 2-3 temples were randomly chosen in each district, and 5-10 stray cats were sampled at each temple.

### Samples and data collection

In total, 473 blood samples were collected from semi-domesticated cats. Each cat was manually restrained, and a blood sample was drawn from the jugular vein using a sterilized syringe. Samples were transferred to Vacutainer tubes containing sodium citrate anticoagulant. All blood samples were taken by a qualified veterinarian or veterinary technician. Each cat was carefully combed for at least 5 min over its entire body surface and inspected for the presence of ectoparasites. Information recorded for each animal (age, gender, free-roaming status, ectoparasites, and deworming) was provided by the monastery caretaker.

### Molecular characterization and DNA sequencing

A commercially available extraction kit (E.Z.N.A.^®^ Blood DNA Mini Kit, Omega Bio-tek Inc., Norcross, Georgia, USA) was used to extract DNA following the manufacturer’s instructions. The final elution volume was reduced to 100 mL, and the DNA was stored at −20°C in a freezer in the Department of Parasitology, Faculty of Veterinary Medicine, Kasetsart University, Bangkok, Thailand, until use. Amplification targeting hemoplasma 16S rRNA used conventional PCR. Briefly, 1 mL DNA was mixed with KAPA2G Fast HotStart ReadyMix with dye (0.4× final concentration) (Kappa Biosystems, Wilmington, Massachusetts, USA) containing 0.08 mM of each dNTP, 0.2 U KAPPA 2G Fast DNA polymerase, 0.6 mM MgCl_2_, 0.5 mL primer (HBT-F) =5’ ATACGGCCCATATTCCTACG 3’ (positions 313–332 in AF-178677), and 0.5 mL primer (HBT-R) =5’ TGCTCCACCACTTGTTCA 3’ (positions 889-908 in AF-178677) [23]. The volume was adjusted to 10 mL using DNase-free distilled water. The amplification was performed using an Eppendorf MasterCycler Nexus Gradient Thermal Cycler (Eppendorf AG, Hamburg, Germany) under the following conditions: Pre-denaturation at 95°C for 5 min, denature at 95°C for 30 s, annealing at 60°C for 30 s, extension at 72°C for 30 s, and a final extension at 72°C for 10 min. Reactions were then incubated at 15°C. The PCR product was checked using electrophoresis on 1.5% agarose gel (LE agarose, Thermo Fisher Scientific, Waltham, USA) and Tris-acetate-EDTA buffer [23]. Product was cut from the gel and purified using a FavorPrep™ GEL/PCR Purification Kit (Favorgen Europe, Shuttleworthstraße, Vienna). Subsequently, the purified product was submitted for Sanger DNA sequencing (Macrogen, Beotkkot-ro, Geumcheon-gu, Seoul, Korea). DNA sequences were proved using the Finch TV version 1.4.0 software (https://digitalworldbiology.com/FinchTV) before phylogenetic analysis.

### Phylogenetic analysis

DNA sequences were compared with previously published sequences of hemoplasmas using the basic local alignment search tool program (https://blast.ncbi.nlm.nih.gov/Blast.cgi) developed by the nation center for biotechnology information. Nucleotide sequences were compared with other sequences submitted to GenBank, and alignment was achieved using the BioEdit program version 7.5.2. Phylogenetic analyses used maximum likelihood analysis with the aid of Mega 7 software (www.megasoftware.net). Bootstrap analyses were based on 1000 iterations.

### Statistical analysis

Sample sizes were estimated using ProMESA software (http://www.promesa.co.nz/ProMESA_Info.htm) with an assumption of standard normal distributions and 95% confidence limits. Statistical associations among results from PCR analysis and categorical variables regarding gender, age, free-roaming status, and location were examined using a Chi-square test (cell frequencies of >5) or Fisher’s exact test (cell frequencies of ≤5) with a 95% confidence interval (95% CI) using R software (version 3.6.1). A logistic regression model was used to test for independent risk factors associated with infection. Multinomial logistic regression and binomial logistic regression were utilized for independent variables with more than 2 groups and independent variables with two groups, respectively. Associations were considered statistically significant when p<0.05. Both p-values and odds ratios (OR) with 95% CI are reported.

## Results

### Characterization of samples

Of 473 semi-domesticated cats studied, 104 were under 1 year old (21.98%), 330 cats were aged 1-5 years (69.76%), and 39 cats were older than 5 years (8.21%). Male cats made up just under one-half of the population (45.66%; 216/573). High proportions of cats were allowed to roam freely in monasteries (91.12%; 431/473), diagnosed with an ectoparasite infestation (48.2%; 228/473), and having not been dewormed (94.08%; 445/473).

### Prevalence of hemoplasma infection

The 16S rRNA gene-based feline hemoplasma DNA was detected in 180 of 473 samples (38.05%). From the 25 districts used for DNA sequencing, 90 positive samples revealed 83.33% (75/90) *CMhm*, 13.33% (12/90) *Mhf*, and 3.33% (3/90) *CMt* infection. Further, in 25 districts positive with hemoplasma, 100% (25/25) districts, 36% (9/25) districts, and 12% (3/25) districts were infected with *CMhm*, *Mhf*, and *CMt*, respectively. *CMhm*+*CMt* (12%) were detected in 3 out of 25 districts including Bueng Kum, Bang Na, and Bangkok Yai, while *CMhm*+*Mhf* (32%) were detected in 8 out of 25 districts, including Khan Na Yao, Bueng Kum, Phaya Thai, Phra Khanong, Rat Burana, Klong Toei, Bangkok Yai, and Chom Thong; *CMt*+*Mhf* (8%) were detected in Bueng Kum and Bangkok Yai; and *CMhm*+*CMt*+*Mhf* (8%) were detected in Bueng Kum and Bangkok Yai.

Feline hemoplasmas was detected in semi-domesticated cats in Bangkok monasteries in 33 out of 34 districts (97.05%). Both Bueng Kum and Bangkok Yai had a predominantly positive result with all three species, while Huai Khwang was negative for hemoplasma infection (Figure-1).

### Risk factors associated with hemoplasma infections

The occurrence of hemoplasma infection was 20-40% (20/104; 132/330) in younger cats (age 1-5 years) and much higher for hemoplasma in animals over 5 years of age (up to 70% [28/39]). This difference was statistically significant (χ^2^=15.147, p=0.0005, OR=3.8, 95% CI=1.64-4.78). Occurrence of hemoplasma infection due to other factors such as gender, free-roaming status, ectoparasites, and deworming in cats was in a range from 37% to 42% (Table-1).

**Table-1:** Prevalence of hemoplasma infection in semi-domesticated cats and risk factors associated with infection.

Factor	Number of cats (%)	Number of positive cats (%)	Chi-square test	Odds ratio (CI)
Age (year)	473	180 (38.05)	c2=15.147, df=2 p-value=0.0005	3.8* (1.64-4.78)
≤1	104 (21.98)	20 (19.23)		
1<-≤5	330 (69.76)	132 (40.00)		
>5	39 (8.21)	28 (71.79)		
Gender	473		c2=0.032, df=1 p-value=0.95	0.67 (0.67-1.41)
Male	216 (45.66)	83 (38.43)		
Female	257 (62.22)	97 (37.74)		
Free-roaming	473		c2=0.25, df=1 p-value=0.61	0.8 (0.42-1.53)
Yes	431 (91.12)	162 (37.59)		
No	42 (8.87)	18 (42.86)		
Ectoparasites	473		c2=2.76, df=1 p-value=1	1.01 (0.7-1.46)
Yes	228 (48.20)	87 (38.15)		
No	245 (51.79)	93 (37.95)		
Dewormed	473		c2=0.54, df=1 p-value=0.45	1.44 (0.67-3.11)
Yes	28 (5.919)	13 (46.43)		
No	445 (94.08)	167 (37.53)		

* Significant difference at p<0.05 with 95% CI. CI=Confidence intervals

### Phylogenetic analysis

**Figure-2:**
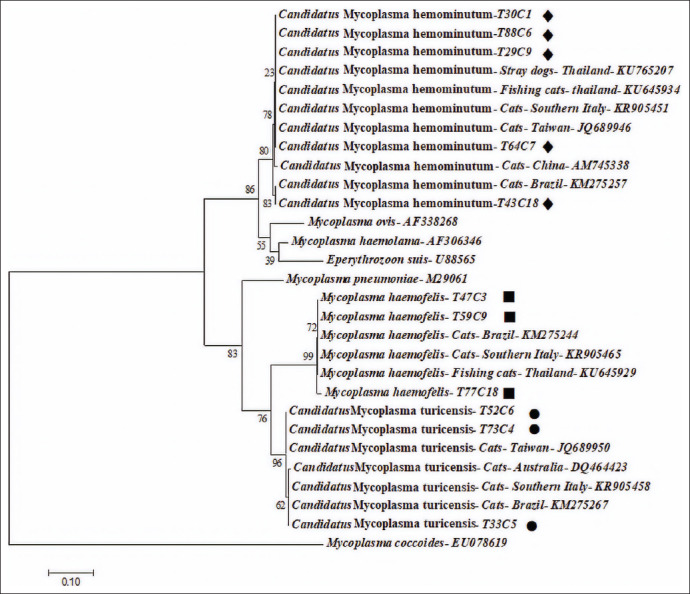
Phylogenetic tree of feline hemoplasma based on the nucleotide sequences of a 595 bp fragment of 16S rRNA gene using the maximum likelihood method. Numbers at nodes represent percentage occurrences of clades based on 1000 bootstrap replications of data. The *CMhm, Mhf*, and *CMt* sequences generated in the present study are in bold and indicated by black diamond (♦), square (◼), and circle (●) symbols, respectively.

Based on the 16S rRNA gene, *CMhm*16S rRNA sequences (approximately 557 – 595 bp in length) were 99.29-99.47%, which is identical to published sequences (GenBank accession numbers KR905451, KU645935, FJ004275, KM275275, and KU765207). *CMt* (approximately 557 – 595 bp in length) showed high sequence identity (99.12%) with published sequences in GenBank (GenBank accession numbers DQ464423, KR905458, KM 275267, and JQ689950). Similarly, *Mhf* sequences (approximately 557 – 595 bp in length) shared 99.29% identity with submitted sequences (GenBank accession numbers KU645929, KR905465, and KM275244). From this study, 30 sequences of hemoplasma obtained and previously published reports were used to establish a phylogenetic tree. Phylogenetic analysis showed three clades of feline hemoplasma: *CMhm* (Clade 1), *CMt* (Clade 2), and *Mhf* (Clade 3) (Figure-2).

## Discussion

All three species of feline hemoplasmas (38.05%, 180/473) were detected in semi-domesticated cats. *CMhm* was the most prevalent species (83.33%). Semi-domesticated cats showed higher rates of infections than owned cats in Bangkok, displaying 22.9% positive incidence by PCR analysis [24]. The high incidence of hemoplasmas in semi-domesticated cats is not surprising since outdoor access is a recognized risk factor for infection based on the previous surveillance [10,17,25]. For example, Willi *et al*. [10] found a significant relationship between outdoor access and hemoplasma infection in client-owned cats in Switzerland (OR=2.7, p=0.01). Free-ranging cats might have more exposure to bloodsucking arthropods and may participate in more aggressive fighting activity than owned cats kept primarily indoors. Both factors can increase infection risk for hemoplasma.

The prevalence of hemoplasmas in the survey (38.05%) was higher than prevalence reported in other studies on stray cats worldwide, where incidence ranged from 9% to 16% [1,15-17,18]. This difference in prevalence may be due to factors such as geographical variation and cat populations sampled (client-owned cats, stray cats, and semi-domesticated cats). A higher prevalence of feline hemoplasma infection is commonly observed in countries with warmer climates [1,17]. Thailand is located in Southeast Asia and experiences a tropical climate throughout the year, which may support a diversity of blood-feeding arthropods compared to more temperate areas. Such arthropods (fleas and ticks) are possible vectors for disease transmission [19]. Further, other suspected modes of transmission, such as biting or other aggressive interactions, may also spread hemoplasmas infection among semi-domesticated cats [6].

In the current study, the prevalence of *CMhm* infection in semi-domesticated cats was higher than other feline hemoplasmas species (*CMt* and *Mhf*), which is consistent with other investigations worldwide [10,25,26]. Tanahara *et al*. [18] reported lower virulence for *CMhm* that allows it to efficiently infect and survive for extended periods in asymptomatic animals. Furthermore, hemoplasmas-infected cats were significantly older than uninfected cats, which are concordant with the previous reports that adult animals are more frequently exposed to bloodsucking arthropods and also have more aggressive interactions, both of which could enhance transmission through infected blood [3,17,27].

Phylogenetic 16S rRNA gene-based analysis shows that our sequences separate into three distinct clades, one for each hemoplasmas species. A previous report identified two major groups of hemoplasmas, including a group encompassing *Mhf* and *CMt* species and a hemominutum group with *CMhm* [28]. Phylogenetic analysis shows that sequences from *Mhf* and *CMt* belong to the same clades as *Mhf* and *CMt* strains from Brazil, Italy, Taiwan, and Australia. Sequences of *CMhm* from semi-domesticated cats show more variation from strains isolated in Italy, Taiwan, China, and Brazil; however, they all belong to the same clade, *CMhm*. Interestingly, based on phylogenetic analysis, *CMhm* in cats in the current study was 99.29-99.47% identical to *CMhm* in stray dogs in Thailand (GenBank accession no. KU765207). This observation may be due to stray cats and dogs residing in the same location and also being exposed to the same pathogens through transmission modes discussed above. Control of hemotropic mycoplasmas in semi-domesticated cats, along with control of other pathogens in stray animals, should be considered by Thai veterinarians, the Thai government, and Thai authorities.

## Conclusion

This survey demonstrates the prevalence and distribution of feline hemoplasmas in semi-domesticated cats. Such information can contribute to developing effective prevention and control strategies to minimize infections by feline vector-borne pathogens. However, further investigation involving sampling of domesticated house cats and the prevalence of hemoplasmas infection in ectoparasites is needed to clarify routes of pathogen transmission and to fully elucidate the prevalence of feline hemoplasmas infections in Thailand.

## Authors’ Contributions

TI planed and designed the experiment and data analysis and revised the manuscript. TD conducted the experiment, interpreted the results, and drafted the manuscript. KK collected the samples and revised the manuscript and LKB revised the manuscript. All authors have read and approved the final manuscript.
